# Molecular Epidemiologic Investigation of an Anthrax Outbreak among Heroin Users, Europe

**DOI:** 10.3201/eid1808.111343

**Published:** 2012-08

**Authors:** Erin P. Price, Meagan L. Seymour, Derek S. Sarovich, Jennie Latham, Spenser R. Wolken, Joanne Mason, Gemma Vincent, Kevin P. Drees, Stephen M. Beckstrom-Sternberg, Adam M. Phillippy, Sergey Koren, Richard T. Okinaka, Wai-Kwan Chung, James M. Schupp, David M. Wagner, Richard Vipond, Jeffrey T. Foster, Nicholas H. Bergman, James Burans, Talima Pearson, Tim Brooks, Paul Keim

**Affiliations:** Northern Arizona University, Flagstaff, Arizona, USA (E.P. Price, M.L. Seymour, D.S. Sarovich, S.R. Wolken, K.P. Drees, S.M. Beckstrom-Sternberg, R.T. Okinaka, W.-K. Chung, J.M. Schupp, D.M. Wagner, J.T. Foster, T. Pearson, P. Keim);; Health Protection Agency, London, UK (J. Latham, J. Mason, G. Vincent, R. Vipond, T. Brooks);; National Biodefense Analysis and Countermeasures Center, Frederick, Maryland, USA (A.M. Phillippy, S. Koren, N. H. Bergman, J. Burans);; and Translational Genomics Research Institute, Phoenix, Arizona, USA (S.M. Beckstrom-Sternberg, J.M. Schupp, P. Keim)

**Keywords:** Anthrax, heroin, canSNP, epidemic, outbreak, Scotland, Bacillus anthracis, epidemiology, Trans-Eurasian, phylogenetic, bacteria

## Abstract

In December 2009, two unusual cases of anthrax were diagnosed in heroin users in Scotland. A subsequent anthrax outbreak in heroin users emerged throughout Scotland and expanded into England and Germany, sparking concern of nefarious introduction of anthrax spores into the heroin supply. To better understand the outbreak origin, we used established genetic signatures that provided insights about strain origin. Next, we sequenced the whole genome of a representative *Bacillus anthracis* strain from a heroin user (Ba4599), developed Ba4599-specific single-nucleotide polymorphism assays, and genotyped all available material from other heroin users with anthrax. Of 34 case-patients with *B. anthracis*–positive PCR results, all shared the Ba4599 single-nucleotide polymorphism genotype. Phylogeographic analysis demonstrated that Ba4599 was closely related to strains from Turkey and not to previously identified isolates from Scotland or Afghanistan, the presumed origin of the heroin. Our results suggest accidental contamination along the drug trafficking route through a cutting agent or animal hides used to smuggle heroin into Europe.

## Abstract 2

Heroin may have been accidentally contaminated by an animal-derived source along a major drug trafficking route.

*Bacillus anthracis* is a gram-positive endospore-forming bacterium that causes the disease anthrax in livestock, wildlife, and humans. Because of its hardy spores, *B. anthracis* can survive for extended periods in the environment, a trait that likely contributed to the successful global spread of this organism (*1*). The mostly dormant life cycle of *B. anthracis* and its relatively recent emergence as a pathogen have resulted in a genome that is highly clonal, with little genetic variation among even the most distantly related strains (*2**–**4*).

Anthrax is most commonly contracted by exposure to contaminated animal products, such as skins, wool, or meat; its symptoms vary in severity depending on the route of infection. Cutaneous anthrax, the most common manifestation of disease, accounts for 95% of cases, whereas pulmonary and gastrointestinal anthrax are much less common and follow inhalation or ingestion of spores, respectively. Inhalational anthrax is rare but particularly deadly, with up to a 90% fatality rate (*5*).

In 2000, a novel form of cutaneous anthrax, termed injectional anthrax, was proposed after anthrax was diagnosed in a heroin “skin popper” (one who injects the drug beneath the skin, rather than into a vein) from Norway on postmortem examination (*6*). Injectional anthrax symptoms are more severe than those of cutaneous anthrax and are typified by severe soft tissue infection at the injection site, which can progress to septic shock, meningitis, and death (*7*). The origin of anthrax in the Norwegian heroin user was never identified, although contaminated heroin was suspected (*6*). This case was the first to demonstrate this previously unrecognized route of *B. anthracis* infection.

In December 2009, two cases of injectional anthrax were diagnosed in heroin users in Scotland after *B. anthracis* was detected in blood cultures (*8*). These cases marked the beginning of an emerging anthrax outbreak among European heroin users. Over the following months, 14 anthrax deaths were confirmed and 119 anthrax cases were suspected (*9*), leading to increasing media attention as the severity of this outbreak became more apparent. This attention was spurred by 3 factors. First, *B. anthracis* is not found naturally in Scotland, and human cases of anthrax in Europe are extremely rare, with only 3 cases of anthrax notified in Europe in 2008 (*10*). Second, the pathology of injectional anthrax is especially devastating (*11**,**12*). Third, anthrax cases appeared to befall only an ostensibly targeted population of persons, leading to initial suggestions of deliberate contamination of the heroin supply. Although investigations were unable to show nefarious intent, the mode of contamination with *B. anthracis* spores remained elusive because of an inability to culture *B. anthracis* from, or detect *B. anthracis* DNA in, suspected contaminated heroin (*9*).

In the current study, we applied a molecular phylogeographic approach to identify the likely origin of the *B. anthracis* spores responsible for the 2009–2010 outbreak in Europe. We used canonical single-nucleotide polymorphism (canSNP) genotyping against heroin anthrax samples and an extensive collection of diverse worldwide samples (*1**,**13*) and whole genome sequencing techniques to determine a possible origin for the *B. anthracis* spores responsible for this outbreak.

## Materials and Methods

### *B. anthracis*–containing Heroin Samples

We obtained 36 samples, positive for *B. anthracis* by culture or PCR, from 34 injecting heroin users throughout the duration of the epidemic (December 2009–November 2010) (Table). Most samples were from users in Scotland (n = 29), with 5 samples from England, 1 sample from Germany, and 1 sample of unknown origin.

**Table:** Genotyping results of 36 culture- or PCR-confirmed cases of anthrax in heroin users, 2009–2010, Europe*

Sample no.	Status†	Collection date	Location	SNP1053700	SNP1173928
Ames‡	NA	1981	Texas, USA	A	G
Ba4599§	C+/PCR+	2009 Dec 16	Glasgow, Scotland	G	C
A112	C+/PCR+	2009 Dec 18	Germany	G	C
4622	C+/PCR+	2009 Dec 19	Glasgow, Scotland	G	C
4646	C-/PCR+	2009 Dec 22	Airdrie, Scotland	G	C
4670	C+/PCR+	2009 Dec 23	Glasgow, Scotland	G	C
4745	C-/PCR+	2009 Dec 31	Glasgow, Scotland	G	C
**0002**	**C+/PCR+**	**2010 Jan 4**	**Glasgow, Scotland**	**G**	**C**
**0007**	**C+/PCR+**	**2010 Jan 4**	**Glasgow, Scotland**	**G**	**C**
0001	C+/PCR+	2010 Jan 4	Dundee, Scotland	G	C
0046	C-/PCR+	2010 Jan 6	Stirling, Scotland	G	C
**0074**	**C+/PCR+**	**2010 Jan 7**	**Kirkcaldy, Scotland**	**G**	**C**
**0075**	**C+/PCR+**	**2010 Jan 7**	**Kirkcaldy, Scotland**	**G**	**C**
0117(2)	C+/PCR+	2010 Jan 9	Dundee, Scotland	G	C
0142	C+/PCR+	2010 Jan 9	Glasgow, Scotland	G	C
0271	C-/PCR+	2010 Jan 15	Glasgow, Scotland	G	C
0393	C+/PCR+	2010 Jan 20	Kilmarnock, Scotland	G	C
0426	C+/PCR+	2010 Jan 21	Glasgow, Scotland	G	C
0491	C+/PCR+	2010 Jan 22	Glasgow, Scotland	G	C
0773	C+/PCR+	2010 Feb 4	London, England	G	C
0871	C+/PCR+	2010 Feb 9	Glasgow, Scotland	G	C
0874	C+/PCR+	2010 Feb 9	Glasgow, Scotland	G	C
0844	C-/PCR+	2010 Feb 9	Blackpool, England	G	C
1297	C+/PCR+	2010 Feb 26	London, England	G	C
1060	C+/PCR+	2010 Mar 5	Fife, Scotland	G	C
1134	C+/PCR+	2010 Mar 5	Dumfries, Scotland	G	C
1320	C+/PCR+	2010 Mar 5	Dumfries, Scotland	G	C
0981	C-/PCR+	2010 Mar 5	Airdrie, Scotland	G	C
1458	C-/PCR+	2010 Apr 1	Dumfries, Scotland	G	C
1927	C-/PCR+	2010 Apr 1	Paisley, Scotland	G	C
2145	C-/PCR+	2010 Apr 16	Livingston, Scotland	G	C
2199	C-/PCR+	2010 Apr 16	Glasgow, Scotland	G	C
2506	C+/PCR+	2010 Apr 27	Edinburgh, Scotland	G	C
2728	C+/PCR+	2010 May 7	Glasgow, Scotland	G	C
3739	C+/PCR+	2010 Jul 16	Glasgow, Scotland	G	C
4936/5011	C-/PCR+	2010 Aug 26	Leicester, England	G	C
6696	C+/PCR+	2010 Nov 1	Maidstone, England	G	C

*ID, identification; SNP, single nucleotide polymorphism; NA, not applicable; C, culture. **Boldface** indicates pairs of isolates from the same patient. †PCR+ samples were determined by using assays targeting *cap*, *lef* (Special Pathogens Reference Unit (UK), pers. comm.) and *bagC* loci (*14*). ‡Ancestral allele DNA control. §Derived allele DNA control.

### DNA Extraction

A *B. anthracis* isolate obtained from a Scottish heroin user in December 2009 (Ba4599) was cultured for 18 hours on 5% sheep blood agar, and genomic DNA was obtained by using phenol/chloroform extraction. All other *B. anthracis* isolates obtained during the outbreak were extracted by using a QIAamp DNA Mini Kit (QIAGEN, Valencia, CA, USA). We extracted 337 geographically diverse *B. anthracis* isolates from the Trans-Eurasian (TEA) group of the *B. anthracis* phylogeny (*1**,**4**,**15*) by using 5% Chelex 100 (Bio-Rad, Hercules, CA, USA) (*16*). Strains for whole genome sequencing were extracted by using the DNeasy Blood and Tissue kit (QIAGEN).

### SNP Genotyping

Several previously established canSNP real-time PCR assays for *B. anthracis* were used to determine the approximate phylogenetic placement of Ba4599 on the *B. anthracis* phylogenetic tree (*1**,**15**,**17*). This approach enabled us to identify a subset of isolates that matched the Ba4599 genotype according to canSNP genotyping. Novel assays within the A.Br.008/009 group were then run against Ba4599 and other A.Br.008/009 samples in our collection to refine the phylogenetic placement of Ba4599 within A.Br.008/009 and to identify its closest known relatives.

To divide the A.Br.008/009 TEA group into 2 lineages (A.Br.008/011 and A.Br.011/009; Figure), we converted the canSNP A.Br.011 assay (*17*) into a TaqMan MGB dual-probe real-time PCR (Applied Biosystems, Foster City, CA, USA). These A.Br.011 primers and probes (5′→3′) were used: ABr011_For: CTAAAAAGAAACGAATTCCCGCTGA, ABr011_Rev: CGATAAAAATCGGAATTGAAGCAGGAG, ABr011_Anc: VIC-CGCCCCCATTATTT, and ABr011_Der: 6-FAM-CGCCCCTATTATTT. The SNP position is underlined in the TaqMan probe sequences.

**Figure:**
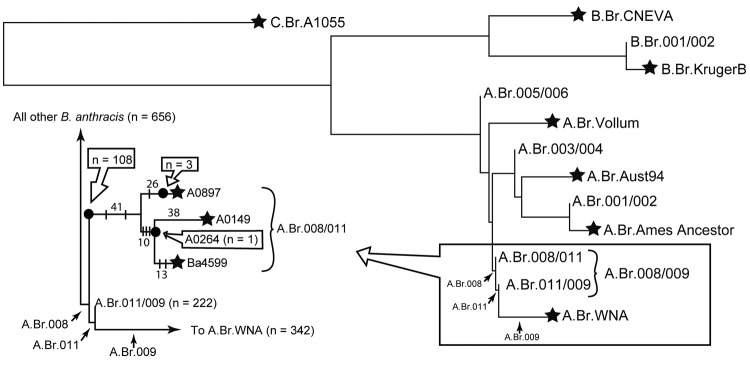
Phylogenetic location of the heroin Ba4599 genome on the global *Bacillus anthracis* phylogenetic tree (*1*). Ba4599 was isolated from a heroin user who died of anthrax at the beginning of the 2009–2010 European anthrax outbreak. Canonical single nucleotide polymorphism (SNP) typing situated Ba4599 within the A.Br.008/011 clade of the Trans-Eurasian group (arrows). Closer examination of the boxed area (inset) revealed that Ba4599 was closely related to 2 isolates from Turkey, A0149 and A0264. Solid black circles indicate the approximate position of collapsed branches (n = 3) and are labeled with the corresponding number of strains that fall within the node. Branch termini are occupied by whole genome sequenced strains (black stars). SNPs are numbered on a given branch; vertical bars along the A.Br.008/011 branches denote the phylogenetic placement of SNP assays used in our analysis of *B. anthracis* from heroin users in Europe. Consistency index = 0.923.

The *B. anthracis* isolate from heroin and 114 other isolates from our collection clustered within the A.Br.008/011 lineage. We therefore focused on further resolving the positions of members of this group. Comparison of the A0897 genome (see below) with previously published genomes (*1**,**3*) yielded 67 SNPs within this lineage (Figure). Three of these SNPs were incorporated into allele-specific real-time PCR assays (*18*) and used to reduce the list of closest relatives to Ba4599 (Figure). The A.Br.008/011 SNP assays are named according to their location on the Ames Ancestor chromosome (GenBank accession no. AE017334) and are listed in order of increasing proximity to the terminal genome on this branch, *B. anthracis* strain A0897. Primers for these 3 A.Br.008/011 lineage SNPs are shown in 5′ → 3′ orientation and as ancestral, derived, and common primers, respectively: SNP5013862 (ATTGAAATGATGATTTTTCACgA, GATTGAAATGATGATTTTTCACgT, TGGTTTATACCATTGTATTGCCCG), SNP1967560 (AATCATCAACATGGTCTTCTGTAAaC, AATCATCAACATGGTCTTCTGTAAgA, GAAAAACCAGAAGTAGTGTGCGGTG), and SNP1118831 (CTCGCTCTGCGTACGTTTG, CTCGCTCTGCGTACGTTcA, TATCAATCTGAAGAAGGTAGCGATAACG). Underlined nucleotides indicate the SNP position, and lowercase nucleotides indicate deliberately incorporated penultimate mismatches for enhanced allele specificity (*19*). These 3 SNPs grouped 2 samples (A0149 and A0264) from our collection with Ba4599.

### Whole Genome Sequencing

We sequenced 3 genomes (Ba4599, A0897, and A0149) to place Ba4599 into a broader phylogenetic context and to accurately establish the evolutionary relationships among these strains. We also sought to identify heroin strain–specific SNPs that could be used to rapidly determine whether *B. anthacis* that caused infections in heroin users was from the same point source as Ba4599. Because of the highly clonal nature of *B. anthracis* and its low mutation rate, phylogenetic analysis using small numbers of SNPs derived from whole genome sequencing is a highly accurate method for determining qualitative and quantitative patterns of relatedness (*3**,**20*). With the exception of A0149 and previously sequenced strains, isolates were subjected to paired-end whole genome sequencing on the Illumina Genome Analyzer IIx instrument (Illumina Inc., San Diego, CA, USA). Additional sequencing was performed for Ba4599 on the 454 GS FLX instrument (454 Life Sciences, Branford, CT, USA) to provide complementary validation of whole genome sequencing SNP calls and improve contiguity of the genome assembly. De novo hybrid assembly of the Ba4599 Illumina and 454 data was performed by using Celera Assembler version 6.1 (*21*). Ba4599 contigs are available in DDBJ/EMBL/GenBank (accession nos. AGQP00000000 and AGQP01000000).

A0149 was sequenced with a shotgun Sanger library approach by using pUC19 and M13 reads before electrophoresis on an AB3730*xl* instrument (Applied Biosystems). Read mapping was performed by using BWA and BWA-SW 0.5.9 (*22**,**23*). Ames Ancestor (GenBank accession nos. AE017334–AE017336) was used as the reference genome for assembly and WesternNA USA6153 (GenBank accession no. AAER00000000) as a related but distinct genome for the TEA group. Processing and data filtering were performed with SAMtools 0.1.12a (*24*) by using an in-house java script that filtered out results, providing <10× coverage. For in-house genomes, SNP calls were then made by using SolSNP version 1.1 with the following alignment limits: minimum coverage of 20, minimum mapping qualities of 20, and a filter call of 0.95. MUMmer 3.20 (*25*) was used to determine SNP calls in public genome sequences.

### Whole Genome Phylogenetic Analysis

Maximum-parsimony phylogenetic trees were inferred by conducting a heuristic search in PAUP* 4.0b10 (*26*) by using the filtered whole genome sequencing SNP data as input. The tree was rooted by using Ames Ancestor as the outgroup because this strain is not a descendant of the TEA group.

### Phylogenetic Placement of Nearest Neighbors

We did not have whole genome sequencing data for isolate A0264 from Turkey. We therefore used canSNP genotyping assays to determine the approximate phylogenetic location of this strain relative to Ba4599 and A0149. CanSNPs along the branch leading to A0149 and Ba4599 were identified from whole genome sequencing data by searching for SNPs with allelic states shared only between these 2 genomes. We designed assays for 3 of these SNPs (Figure). The positions of these SNPs in the Ames Ancestor genome (NC_007530.2) are listed along with flanking sequence from Ames Ancestor. The SNP is underlined and the derived allele, found in Ba4599, is included in parentheses: SNP1530761 GGGCATTAGGATCAGCGATAA (T), SNP3287006 AGGTTGCCTTCCCCATCTATT (A), and SNP3836105 AATCGTAAAGTGGCTGTATTT (C). Primers for the first SNP (1530761) assay are listed in order of derived, ancestral, and common primers as follows (5′ → 3′): SNP1530761 (CTACTGCTTCTTACACATTTATCGCTGtA, TACTGCTTCTTACACATTTATCGCTGtT, GTTCCGCTCGGTACGGTATC). This assay was the most robust of the 3 tested assays.

We then used whole genome comparisons to identify SNPs along the branch leading to the Ba4599 genome. These SNPs were tested against A0264 and all 36 clinical samples from the heroin outbreak. We targeted 2 of the 13 identified SNPs (Figure) for TaqMan assay design as follows: SNP1053700 (CCTCGGAAATGAAGTGGTTGAAAAT, CGGGAATGTTGACATTAAGCTCATT, VIC-CTGCATAAATACCAGATAGTAA, FAM-CTGCATAAATACCAGGTAGTAA) and SNP1173928 (GCAGGTCTTCGAATGATGTGTCAAT, GCTCTTCCACGATTTCAAAGTCATT, VIC-CCTGTTGTAGAATATCT, FAM-CTGTTGTACAATATCT). Ninety-three TEA isolates unrelated to the heroin outbreak and 2 non-TEA isolates belonging to the Ames (A0462) and Western North America (A0303) groups were screened to confirm specificity of these 2 SNP assays for the Ba4599 heroin genotype.

## Results and Discussion

### Phylogeographic Placement of *B. anthracis* Heroin Strain Ba4599

Anthrax is considered to be endemic to many parts of western, central and southern Asia, with large numbers of human and animal anthrax cases being reported annually in countries such as Afghanistan, Bangladesh, India, Iran, Kazakhstan, Kyrgyzstan, Pakistan, Turkey, and the Republic of Georgia. Molecular epidemiologic studies of *B. anthracis* have demonstrated that particular genotypes correspond with geographic regions, a trait made possible by the relatively recent emergence of this highly clonal pathogen (*1**,**3**,**4*). Therefore, by establishing the genotype of Ba4599, we expected to find clues regarding the geographic origin of the anthrax outbreak caused by contaminated heroin.

Afghanistan produces 90% of the world’s heroin (*27*), and the Khyber Pass region of Pakistan and Afghanistan has historically been one of the major centers for staging and trafficking of heroin into western Europe (*28*). It was therefore logical to suspect that heroin trafficked into Europe during the 2009–2010 anthrax epidemic originated in Afghanistan, having become contaminated at the primary source (*7*). Indeed, some media reports speculated that the potential source of anthrax spores in the heroin supply was a cutting agent derived from *B. anthracis*–infected Afghan livestock (*29*).

We sought to phylogenetically place the representative Ba4599 heroin isolate by using previously established (*1**,**15*) and novel canSNP signatures, thereby pinpointing the potential geographic origin of contamination. Our canSNP typing grouped Ba4599 within the A.Br.008/011 node of the TEA group (Figure). TEA strains are the most common and widespread group of *B. anthracis* in the world, being found in Europe, the People’s Republic of China, Russia, Kazakhstan, and West Africa (*1*). Seventeen recent isolates from animals in Afghanistan were analyzed and proved not to be members of TEA but rather were affiliated with the evolutionarily distinct Vollum clade. These results are inconsistent with the conclusion that heroin became contaminated during primary production and transformation of opium in Afghanistan.

Precise source attribution of the outbreak caused by *B. anthracis*–contaminated heroin is complicated by the wide phylogeographic spread of the TEA group. To improve phylogenetic resolution and to deduce the closest relatives of Ba4599, we identified and characterized additional molecular targets within the A.Br.008/011 group (SNPs 5013862, 1967560, and 1118831). Ba4599 has derived alleles for the first 2 SNPs but an ancestral allele for 1118831; we were therefore interested in identifying other strains with the same canSNP profile as Ba4599. We tested these 3 SNP signatures across all 120 A.Br.008/011 strains within our *B. anthracis* collection, comprising isolates from Albania (n = 5), Argentina (1), Austria (1), China (47), Ethiopia (1), Hungary (3), Iran (1), Italy (21), Norway (1), Pakistan (2), Poland (1), Republic of Georgia (5), Russia (2), Scotland (12), Slovakia (2), Turkey (6), Ukraine (1), or unknown origin (8). Notably, Ba4599 was indistinguishable from only 2 isolates, A0149 and A0264, both of which originated from Turkey. Twelve isolates linked to a fatal case of anthrax in a drum maker in Scotland in 2006, which originated from contaminated animal skins from Ghana, did not match the Ba4599 genotype. Similarly, the bioweaponized *B. anthracis* used on Gruinard Island, Scotland, during World War II (*30*) belongs to the unrelated Vollum clade and is thus distinct from Ba4599.

### Differentiation of Ba4599 and Turk A0149

Although Ba4599 and A0149 are closely related, they are not identical. By using whole genome sequencing, we identified 51 SNPs that differentiated Ba4599 from A0149, of which 12 were unique to Ba4599 and 38 unique to A0149 (Figure). A second isolate from Turkey, A0264, was tested against 3 of the 10 SNPs leading to the A0149/Ba4599 group (SNPs 1530761, 3836105, and 3287006) and the 2 Ba4599-specific SNPs (1173928 and 1053700) to approximate its phylogenetic location. Our analyses of SNPs 1530761, 3836105, and 3287006 revealed that A0264 shared the derived genotype with A0149 and Ba4599. In contrast, A0264 did not match the 2 SNPs along the Ba4599 branch, similarly to A0149 (Figure). Therefore, A0264 probably does not fall on the branch leading to Ba4599; however, precise placement would require assaying all 13 SNPs along the Ba4599 branch or whole genome sequencing of A0264. Four other isolates from Turkey in our collection fall on the A.Br.008/011 branch but possess an ancestral allele for SNPs 1530761, 3836105, and 3287006, and are thus not as closely related to Ba4599 as are A0149 and A0264.

### Source Attribution of *B. anthracis* in Heroin

On the basis of our molecular typing results, we strongly suspect that *B. anthracis* spores were accidentally introduced into the heroin supply in Turkey (or surrounding regions) before being smuggled into Europe. Heroin produced in Afghanistan is thought to be trafficked through 2 major routes: the Silk Route and, more commonly, the Balkan Route. Both of these routes pass through numerous countries where anthrax is endemic and where isolates belonging to the TEA group have been found. Although we were unable to exhaustively test *B. anthracis* isolates along these routes to find a precise match to Ba4599, Turkey is definitely a possible origin, given that this country is central to the heroin smuggling trade from Afghanistan into Europe along the Balkan Route (*31*), and Turkish laboratories are believed to play a key role in conversion of the morphine base into its usable heroin form (*32*).

Thus, our genotyping results support the hypothesis that the heroin was contaminated along the trafficking route and not at its origin (Afghanistan) or destination (Scotland). How it was contaminated is highly speculative, but it may have involved the addition of an animal-derived cutting agent, e.g., bone meal, or, alternately, wrapping in animal hide contaminated with *B. anthracis* spores. Contaminated animal hide products have been implicated in several allochthonous anthrax cases in Europe and the United States (*33**–**38*), including *B. anthracis* isolates from drum skins linked to a 2006 anthrax case in Scotland. Isolates in our collection from other countries along the Balkan and Silk Routes, including Albania, Hungary, Italy, Poland, Russia, and Ukraine, did not match the Ba4599 genotype, giving greater credence to our hypothesis of Turkey as the point of origin. However, we do not possess isolates from several other anthrax-endemic countries along the Silk and Balkan Routes and therefore cannot rule out the possibility of contamination elsewhere in the region.

### Genotypes of Subsequent *B. anthracis* Isolates from Heroin Users

Two Ba4599-specific SNP assays were used to rapidly screen 35 other *B. anthracis*–positive (by culture or PCR) samples from the heroin-associated outbreak, including an isolate obtained from a heroin user with anthrax in Germany (*14*). These 2 SNP assays were also screened across a panel of 92 TEA and 2 non-TEA *B. anthracis* samples to determine assay specificity. All outbreak isolates obtained for this study shared the 2-SNP Ba4599 genotype, suggesting that the outbreak originated from a batch of heroin that was contaminated with *B. anthracis* spores from a single point source. In contrast, none of the nonoutbreak samples matched the Ba4599 genotype at either SNP, demonstrating that these SNPs were specific to Ba4599. The German patient had no history of travel to the United Kingdom, indicating acquisition of anthrax from a single batch of heroin that was disseminated to at least 3 European countries (Scotland, England, and Germany). It is therefore possible that future cases of anthrax may arise, should the contaminated batch of heroin recirculate in the user population.

## Conclusions

In this study, we used a high-resolution molecular epidemiologic approach to identify the geographic source of anthrax spores responsible for the largest outbreak of injectional anthrax observed to date. Using all available clinical material, we found that the 2009–2010 anthrax outbreak that affected heroin users across Scotland, Germany, and England was caused by a single anthrax strain. Further, genetic and genomic analyses demonstrated that the anthrax spores, while not an exact match, were most closely related to isolates from Turkey. Turkey is a central country along the Balkan Route, a common route for trafficking heroin from its primary source in Afghanistan into European countries. Given the commonality of the TEA group throughout the world, and the close relationship of Ba4599 to isolates from Turkey, we do not possess any evidence to suggest that the heroin was contaminated with nefarious intent. Our results suggest accidental contamination from an animal-derived source, such as bone meal (a cutting agent used to dilute heroin) or animal hides. Although no new cases of injectional anthrax have been reported since a case was diagnosed in November 2010 in Maidstone, England, this outbreak may indicate an emerging infectious disease in drug addicts in Europe and that illicit drugs such as heroin are a novel point of entry for this pathogen into non–anthrax-endemic regions. Educating users of the potential risks of acquiring anthrax from their drug use, coupled with maintained vigilance of public health investigators toward identifying anthrax cases, would help mitigate the public health effects of future outbreaks in this susceptible population.
